# Transcriptome Analysis of *Spartina pectinata* in Response to Freezing Stress

**DOI:** 10.1371/journal.pone.0152294

**Published:** 2016-03-31

**Authors:** Gyoungju Nah, Moonsub Lee, Do-Soon Kim, A. Lane Rayburn, Thomas Voigt, D. K. Lee

**Affiliations:** 1 Department of Plant Science, Research Institute of Agriculture and Life Sciences, College of Agriculture and Life Sciences, Seoul National University, 599 Gwanangno, Gwanakgu, Seoul 08826, Republic of Korea; 2 Department of Crop Sciences, University of Illinois at Urbana-Champaign, 1120 S. Goodwin Ave, Urbana, Illinois 61801, United States of America; Louisiana State University Agricultural Center, UNITED STATES

## Abstract

Prairie cordgrass (*Spartina pectinata*), a perennial C_4_ grass native to the North American prairie, has several distinctive characteristics that potentially make it a model crop for production in stressful environments. However, little is known about the transcriptome dynamics of prairie cordgrass despite its unique freezing stress tolerance. Therefore, the purpose of this work was to explore the transcriptome dynamics of prairie cordgrass in response to freezing stress at -5°C for 5 min and 30 min. We used a RNA-sequencing method to assemble the *S*. *pectinata* leaf transcriptome and performed gene-expression profiling of the transcripts under freezing treatment. Six differentially expressed gene (DEG) groups were categorized from the profiling. In addition, two major consecutive orders of gene expression were observed in response to freezing; the first being the acute up-regulation of genes involved in plasma membrane modification, calcium-mediated signaling, proteasome-related proteins, and transcription regulators (e.g., MYB and WRKY). The follow-up and second response was of genes involved in encoding the putative anti-freezing protein and the previously known DNA and cell-damage-repair proteins. Moreover, we identified the genes involved in epigenetic regulation and circadian-clock expression. Our results indicate that freezing response in *S*. *pectinata* reflects dynamic changes in rapid-time duration, as well as in metabolic, transcriptional, post-translational, and epigenetic regulation.

## Introduction

Prairie cordgrass (*Spartina pectinata* Link) is a perennial, rhizomatous, C_4_ grass native to the North American prairie [1,2]. It commonly grows in wet tallgrass prairies, is salt tolerant, and is successfully used in riparian plantings and stream-bank stabilization [3,4]. In addition, it is being considered as a dedicated energy crop for marginal lands due to its abiotic environmental stress tolerances and high biomass yield potential [5–10].

Prairie cordgrass may be a model crop for studying tolerances to stressful environments. It performs well when grown in cold, wet, and saline soils [6,11,12]. Moreover, as the most northerly distributed C_4_ grass species, prairie cordgrass performs comparably to cold-acclimated C_3_ grasses [8]. Even though prairie cordgrass has not been considered to be a major crop until recently, it could have significant impact on crop production in the future because it can grow on marginal land and may provide important information on freezing tolerances of warm-season energy- and food-crop grasses.

Undoubtedly, abiotic stresses have great influence on food production in many areas of the world and also threaten crop-production sustainability [13–15]. Abiotic stresses have caused extensive reductions in plant growth and production and have reduced the value of most major plants and crops by more than 50% [16–18]. Only 10% of the world’s arable land is classified as free of stress [19], while 20% of land is under some kind of mineral stress, 26% is affected by drought stress, and 15% by freezing stress [17]. To increase crop productivity and mitigate food crisis without expanding cultivated lands, it is fundamental to understand the effects of abiotic stress on plants and crops tolerant of stressful environments.

While improving crop yields using conventional plant breeding techniques has been successful [15,20], this approach may now be too slow to meet the demands of an increasing world population given that global climate change exacerbates the frequency and severity of abiotic constraints [21]. Consequently, the detection and exploitation of traits that control the adaptive response of crops to abiotic stresses is a prerequisite for cost-effective applications of genomic-based approaches to breeding for sustainable and stabile yields under adverse conditions [21]. In addition, the development of next-generation sequencing technologies makes it possible to re-sequence entire plant genomes more efficiently and to estimate gene expression at the transcriptome level [22]. Understanding transcriptome dynamics is critical for identifying gene function and phenotypic variations that result from the combination of genotypic and environmental factors.

In previous transcriptomic studies, *Arabidopsis thaliana* (mouseear cress), *Oryza sativa* (rice), *Triticum aestivum* (wheat), *Brassica juncea* (brown mustard), and *Lilium lancifolium* (wild lily) were exposed to cold or sub-zero temperature for several hours to several days to investigate changes in gene expression levels [23–28]. Hundreds to thousands of genes were up- and down-regulated in response to low temperature. Included in these studies were differentially expressed genes involved in signal transduction (receptor kinase, protein kinase/phosphatase, Ca^2+^-binding protein) and transcription factors (TFs) (MYB, WRKY, AP/ERBEP, CRF) that control gene expression and effector molecules (e.g., osmolytes, anti-freezing protein, dehydrin, chaperone, ROS-scavenger enzyme, and ice recrystallization inhibition protein) [29–31]. Some of these genes were successfully transferred to cold or freezing-sensitive crops, such as rice, to improve yields under adverse growing condition [32–34].

Although many low-temperature stress tolerance studies have been conducted, greater understanding of the molecular mechanisms that occur at very early stages of stress are needed to identify stress-perceiving or other molecular components (e.g., epigenetic-related components). Even though prairie cordgrass has unique freezing stress tolerance, little is known about its stress-tolerance mechanisms and transcriptome dynamics. In this analysis, we exposed prairie cordgrass to freezing stress for 5 min and 30 min to investigate its gene expression dynamics.

## Materials and Methods

### Ethics statement

No specific permits were required for the described field study. No specific permissions were required for this location and activities. The location is not privately owned or protected in any way. The field studies did not involve endangered or protected species.

### Plant material

A natural tetraploid (2n = 4x = 40) prairie cordgrass population, PC17-109, was chosen for this research because of its [35] early spring emergence and higher freezing and salt tolerances compared with other cordgrass populations [10]. In addition, the PC17-109 population is late-flowering, develops a phalanx-type rhizome system, and has biomass yields comparable Kanlow switchgrass (*Panicum virgatum* ‘Kanlow’), a high-yielding cultivar [10,36].

Rhizomes from a PC17-109 prairie cordgrass population were collected from a field nursery at the University of Illinois Urbana-Champaign (UIUC) Energy Farm (Urbana, IL, USA) in 2013, and planted into pots to mature. After a year of greenhouse growth, the 5-cm tall plants were transplanted into pots (9×9×12 cm) using Sunshine Metro-Mix^®^950 (Sun Gro Horticulture Distribution Inc., MA, USA) as the growing medium. The grasses were grown in a UIUC greenhouse maintained at 27°C/16°C day/night temperature with 14 h photoperiod providing 400μmol m^-2^s^-1^ photon flux at the plant canopy level. Before the freezing test, 4-week old plants having the first leaf fully expanded were transferred into a controlled-environment chamber (Conviron E15; Controlled Environments, Winnipeg, Manitoba, Canada) under 400μmol m-2s-1 photon flux, 70% humidity, and 27°C/16°C day/night temperature to avoid confounding of any undetected effects from different environment. After 2 weeks, the whole plants were exposed to freezing temperatures.

### Freezing treatments and electrolyte leakage assay

The freezing experiment was conducted using a method described by Friesen et al. [8] with little modification. In order to determine a target freezing temperature and duration tolerance of cordgrass compared to other C_4_ species, seeds from two cordgrass populations (‘IL102’ and ‘PC17-109’), two switchgrass cultivars (‘Kanlow’ and ‘Alamo’), and a commercial corn hybrid (*Zea may* ‘VT3’) were tested. All seeds were surface sterilized using commercial bleach (5.25% hypochlorite) for 20 min, and then rinsed three times with distilled water. After sterilization, seeds were placed on petri dishes. Three seedlings were transplanted into a pot (9×9×12 cm) filled with Sunshine Metro-Mix^®^950 (Sun Gro Horticulture Distribution Inc., MA, USA) and grown in a UIUC greenhouse at 16h day length with light supplementation and 27°C/16°C day/night temperature. When 2–3 fully expended leaves were present, the potted seedlings were placed in a growth chamber (Conviron E15; Controlled Environments, Winnipeg, Manitoba, Canada) under 400μmol m^-2^s^-1^ photon flux, 70% humidity, and 27°C/16°C day/night temperatures for 2 weeks of acclimation. The freezing experiments were conducted at -3°C, -5°C, and -7°C for 0 min, 30 min, 60 min, 90 min, 120 min, 150 min, and 180 min, with three replications.

Based on the results of this preliminary work, the freezing temperature and sampling time were optimized (S1 Fig). When exposed to -7°C, the prairie cordgrass exhibited severe leaf tissue damage within 5 min and death within 30 min, while at -5°C, there was no of significant freezing damage until 60 min when electrolyte leakage started. Therefore, we selected the -5°C exposure for 0, 5 and 30 min with three biological replicates. For each test temperature and time, one replicate consisted of three plants in a pot and the experiment was repeated three times. At each time point, leaf tissues were collected, immediately submerged in liquid nitrogen, and stored at -80°C for later RNA extraction.

After exposure to the freezing temperature, fully expanded leaf tissue was collected from each plant at the time described and assayed for electrolyte leakage. Two cm^2^ of leaf tissue was incubated for 24 hours in 15ml distilled H_2_O at room temperature, and then initial electrolyte leakage (I_EL_) was measured. Total electrolyte leakage (T_EL_) was calculated after autoclaving the vials at 120°C for 30 min. The tissue was then allowed to cool to room temperature. The percentage electrolyte leakage (EL%) was evaluated as EL% = I_EL_ / T_EL_ × 100%.

### Total RNA isolation, library construction, and sequencing

Total RNA was extracted from the leaf tissues using the RNeasy Plant Mini kit (Qiagen, Valencia, CA, USA), following the manufacturer's recommended protocols. The quality and integrity of RNA samples were checked using Pico bioanalyzer chips (Agilent 2100 Bioanalyzer, Santa Clara, CA, USA) at the Keck Center for Comparative and Functional Genomic, UIUC. Libraries of cDNA for RNA-seq were constructed using TruSeq Standard RNA-seq Sample Prep Kit (Illumina, San Diego, CA, USA) according to the manufacturer’s protocol. These cDNA libraries were then sequenced via Illumina HiSeq 2500 platform by the Keck Center. All sequencing reads were deposited in the National Center for Biotechnology Information (NCBI) (8600 Rockville Pike, Bethesda, MD USA 20894) under the SRA accession of SRP066155 (BioProject ID of PRJNA301660; Run ID of SRR2890259).

### *De novo* assembly, expression quantification, and annotation

The overall bioinformatics process was described in S2 Fig. Sequence quality was verified using FastQC v0.10.0. (http://www.bioinformatics.babraham.ac.uk/projects/fastqc/). Trimmed reads were assembled using the Trinity program (https://github.com/trinityrnaseq/trinityrnaseq/), which was also used for the *de novo* transcriptome assembly that combined read sequences of a certain amount of overlap to form longer fragments without N gaps, called contigs. For elimination of redundancy, these contigs were clustered based on contig-sequence similarities using CDhit-est (http://weizhongli-lab.org/cd-hit/) with sequence identity cutoff 0.8. To determine the number of high quality reads (Q>30) that are mapped back to assembled contigs, Bowtie2 (version 2.2.5; http://sourceforge.net/projects/bowtie-bio/files/bowtie2/2.2.5/) was used. Next, cDNA prediction was performed using TransDecoder (https://transdecoder.github.io/), which obtained a total of 44,465 contigs as ORF-containing transcripts and translated peptide sequences. Using the 44,465 contigs, annotation was performed using protein databases from *Arabidopsis thaliana* (TAIR10_pep_20101214; https://www.arabidopsis.org/download/), as a dicot model plant, *Oryza sativa* (Rice pseudo-molecules version 7; ftp://ftp.plantbiology.msu.edu/pub/data/Eukaryotic_Projects/o_sativa/annotation_dbs/), as a monocot model plant, *Sorghum bicolor* (*Sorghum bicolor* version 3.1 DOE-JGI; https://phytozome.jgi.doe.gov/pz/) as a grass known to be a close phylogenetic relative of prairie cordgrass [37], and Plant Protein RefSeq Database (ftp://ftp.ncbi.nlm.nih.gov/refseq/release/plant/) using BLASTX with e-value ≤ E^-10^. Gene ontology analysis was performed using DAVID (https://david.ncifcrf.gov/) with Fisher's exact test (p<0.05) to assign the contigs to three GO terms, "cellular component," "molecular function," and "biological process."

### FPKM estimation and DEG analysis

The contigs were processed for read alignment and abundance estimation with Bowtie (http://bowtie-bio.sourceforge.net/index.shtml) and RSEM (RNA-Seq by Expectation Maximization, http://deweylab.github.io/RSEM/). The expression level of each contig was calculated using the fragments per kilo base of exon per million mapped fragments (FPKM) method, which excluded sequencing discrepancies in the calculation of gene expression and the influence of different gene lengths. The FPKM values of all unigene contigs were measured using the RSEM package. For identification of differentially expressed genes (DEGs) among the three time courses using the log_2_FPKM value, a one-way ANOVA was performed at P<0.05 using JMP 11 (SAS Institute, Cary, NC, USA). After the first round of DEG identification, FDR of < 0.05 and the absolute value of log_2_ (fold change) >1 was used for thresholds to determine significantly different gene expression for the final DEG set. Based on expression patterns, DEGs were categorized using JMP 11(SAS Institute, Cary, NC, USA). The comparison of mRNA expression levels between RNA-Seq and qRT-PCR was evaluated using Pearson correlations computed by SAS (SAS Institute, Cary, NC, USA).

### Quantitative real time PCR analysis

The mRNAs extracted from the same leaf tissues used for RNA-sequencing were used for qRT-PCR validation. First-strand cDNA was synthesized from one microgram of total RNA and oligo(dT) primer using SuperScript^™^ II reverse transcriptase (Invitrogen, Carlsbad, CA, USA) following the manufacturer’s instructions. The qRT-PCR was performed using a StepOnePlus real-time PCR system (Applied Biosystems, Forster City, CA, USA) in a final volume of 20μl including 1μl of cDNA 5μl of H_2_O, 2ul of forward and reverse primers, and 10μl of Power SYBR green PCR master mix (Applied Biosystems, Forster City, CA, USA). Power SYBR green PCR master mix was used to detect the expression of selected genes amplified with primers (S1 Table) designed using Primer 3 software (http://simgene.com/Primer3). The PCR cycling conditions were conducted by incubation at 95°C for 1 min followed by 40 cycles of 95°C for 15 sec and 60°C for 1 min. Dissociation-curve analysis was carried out after the program was completed to confirm the amplification specificity. Three technical replicates were performed for each biological sample. A relative mRNA level was calculated by the relative quantification method (ΔΔC_T_), using the *Actin* gene as an endogenous control.

## Results

### Physiological effect of sub-zero treatment across time course

To estimate the physiological damage resulting from sub-zero treatment, electrolyte leakage was measured and showed that as temperature decreased, freezing damage increased and small changes occurred depending on the plant species. Compared to controls (S3A Fig), leaf electrolyte leakage between prairie cordgrass and switchgrass differed under 2 hr exposure at -3°C, -5°C, and -7°C (S3B Fig). Both prairie cordgrass and switchgrass had less leakage than maize at -3°C. Electrolyte leakage to -5°C in switchgrass differed from that of prairie cordgrass. Electrolyte leakage rapidly increased to 60% in switchgrass, but did not change in prairie cordgrass and maintained the 30% level. Under -7°C treatment, electrolyte leakage increased in both prairie cordgrass and switchgrass (S4 Fig).

### *De novo* assembly, annotation, and GO categorization

Using a total of 385,173,502 Illumina raw reads from the control and freeze-treated leaf tissues, 169,169 contigs were initially assembled. To validate the *de novo* assembly, we mapped high quality (Q>30) reads to the assembled contigs. Of the initial 385,173,502 raw reads, 305,565,748 reads were mapped back to the assembled contigs with mismatch ratios of less than 2%, resulting in 79.3% of the mapping ratios. Of these, there were 44,465 contigs with validated open reading frames that we used for further analysis. The average contig length was 859.50bp, and N50 length was 1,098bp (Table 1).

**Table 1:** Assembly statistics of *S*. *pectinata* leaf transcriptome.

Total number of reads	385,173,502
**Total number of contigs**	44,465
**Maximum contig length (bp)**	16,176
**Average contig length (bp)**	859.5
**Median contig length (bp)**	627
**N50 length (bp)**	1,098
**(A+T)s**	47.51
**(G+C)s**	52.49

**Fig 1:**
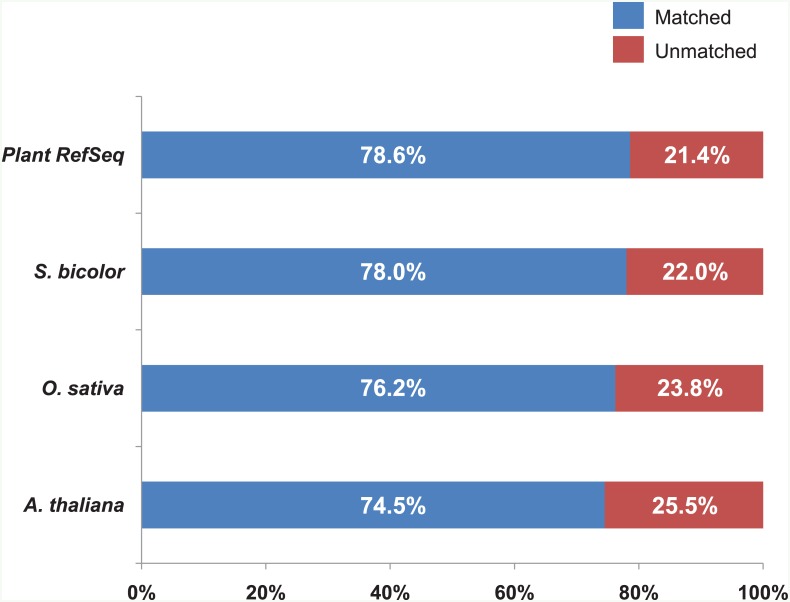
Percentage of homologs in *Spartina pectinata* leaf transcriptome after BLASTX search against the protein databases of *Arabidopsis thaliana*, *Oryza sativa*, *Sorghum bicolor*, and NCBI Plant RefSeq protein with e-value ≤ E^-10^.

To estimate the number of contigs that matched known protein databases, we performed BLASTX against the dicot, C_3_ monocot, C_4_ monocot models, and the total Plant Protein RefSeq database from NCBI. The 44,465 contigs in *Spartina pectinata* transcriptome were translated and blasted against four protein databases: 74.5% (33,146 contigs) against the dicot model (*Arabidopsis thaliana*) protein database; 76.2% (33,885 contigs) against the C_3_ monocot model (*Oryza sativa*) protein database; 78.0% (34,694 contigs) against the C_4_ monocot model (*Sorghum bicolor*) protein database; and 78.6% (34,939 contigs) against the total Plant RefSeq database using BLASTX with e-value ≤ E^-10^ (Fig 1 and S2 Table), suggesting that the majority of *Spartina pectinata* transcriptome was homologous to pre-existing protein databases.

### Gene ontology categorization

**Fig 2:**
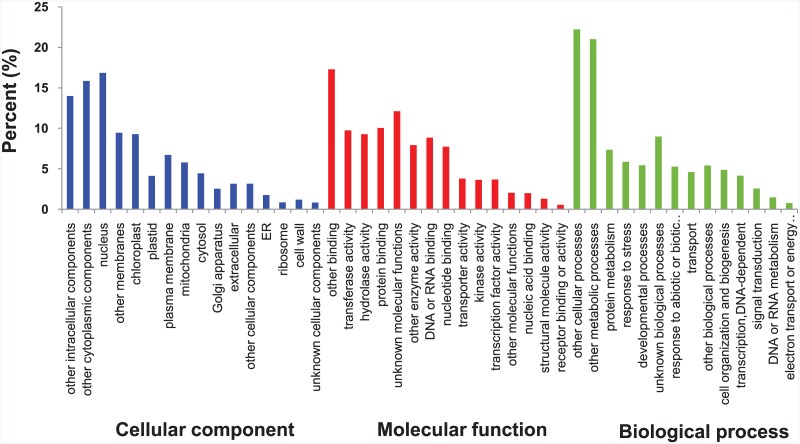
Gene Ontology (GO) analysis of *Spartina pectinata* leaf transcriptome under freezing treatment. The contigs from the *S*. *pectinata* leaf transcriptome were assigned to three GO categories with 16 GO terms of cellular component, 15 GO terms of molecular function, and 14 GO terms of biological process. The x-axis indicates GO terms belonging to three GO categories and the y-axis represents the percentage of contig number in *S*. *pectinata* leaf transcriptome.

To investigate the transcriptome composition, the annotated contigs were subjected to gene ontology (GO) for three categories (cellular process, molecular process, and biological process) (Fig 2). The top six GO terms in the cellular process were ‘nucleus’ (16.83%), ‘other cytoplasmic components’ (15.85%), ‘other intracellular components’ (13.98%), ‘other membranes’ (9.45%), ‘chloroplast’ (9.28%), and ‘plasma membrane’ (6.70%). Next, the top six GO terms in the molecular process were ‘other binding’ (17.29%), ‘unknown molecular functions’ (12.11%), ‘protein binding’ (10.04%), ‘transferase activity’ (9.74%), ‘hydrolase activity’ (9.27%), and ‘DNA or RNA binding’ (8.85%). Finally, the top six GO terms in the biological process were ‘other cellular processes’ (22.22%), ‘other metabolic processes’ (21.02%), ‘unknown biological processes’ (8.99%), ‘protein metabolism’ (7.35%), ‘response to stress’ (5.86%), and ‘developmental processes’ (5.44%).

### Six groups of DEGs in response to freezing treatment

**Fig 3:**
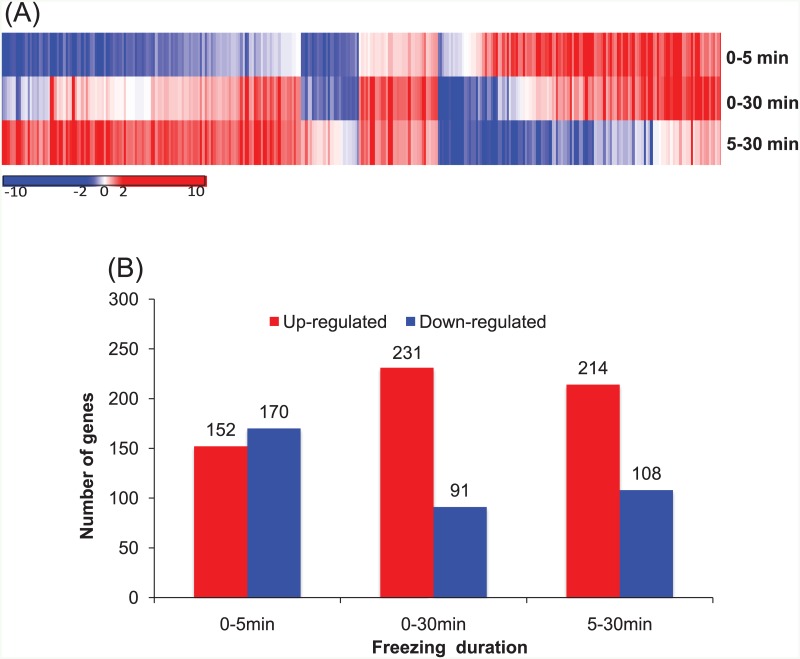
Analysis of differentially expressed genes. (A) Hierarchical clustering (Pearson correlation with average linkage method) of 322 DEGs from 0 min vs. 5 min (0–5 min), 0 min vs. 30 min (0–30 min), and 5 min vs. 30 min (5–30 min) in pair-wise comparisons. Red is up-regulation, and blue is down-regulation. The color bar indicates the range of maximum and minimum values of log_2_ fold change of FPKM. (B) The number of up- and down-regulated genes belonging to 322 DEGs in pair-wise comparisons of three treatments (0 min, 5 min, and 30 min).

To investigate gene expression changes, we calculated the normalized FPKM (Fragments per kilobase per million) value and performed hierarchical clustering using Pearson's correlation. There were 322 significant DEGs (q < 0.05 and |log_2_ (fold change)| > 1) in the freezing treatments (Fig 3A and S3 Table). The pair-wise comparisons among the three time courses (0–5 min; 5–30 min; 0–30 min) are shown in Fig 3B. Of these, the 0–5 min comparison showed a similar number of up- and down-regulated genes (152 up-regulated and 170 down-regulated in 5 min) compared to the 0–30 min (231 up-regulated and 91 down-regulated in 30 min) and 5–30 min (214 up-regulated and 108 down-regulated in 30 min) treatments.

**Fig 4:**
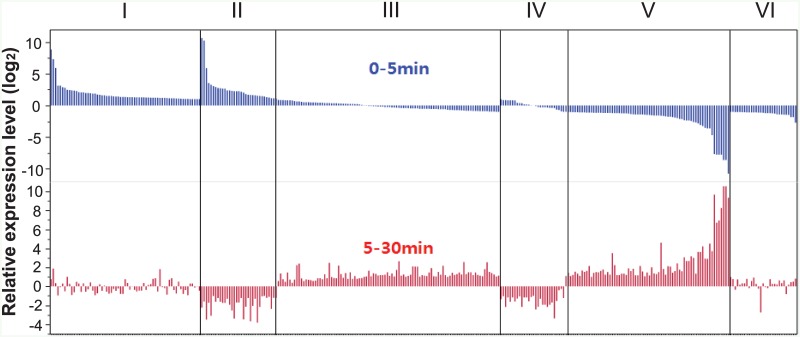
Six groups of relative gene expression change of 322 DEGs. The gene expression pattern change between two phases, 0–5 min and 5–30 min, were classified into six groups (I, II, III, IV, V, and VI).

We divided the expression change patterns into six major groups based on regulation and log_2_ fold change: (i) up-regulation in 5 min and no significant change (|log_2_ (fold change)| < 1) until 30 min, (ii) up-regulation in 5 min followed by down-regulation in 30 min, (iii) no significant change by 5 min, but up-regulation in 30 min, (iv) no significant change by 5 min and down-regulation in 30 min, (v) down-regulation in 5 min followed by up-regulation in 30 min, and (vi) down-regulation in 5 min with no significant change until 30 min (Fig 4). Our investigation focused on regulatory genes encoding transcription factors (TFs) and chromatin-modification proteins, as well as on abiotic stress-related genes, including signal transduction components.

**Group I**: Group I was characterized by an acute up-regulation response within 5 min and no significant increase afterward. This group was composed of 65 genes (S3 Table), including seven TF genes with homologs in *Oryza sativa*, encoding MYB42 (Os09g0532900), WRKY74 (Os09g0334500), GATA21 (Os02g0220400), AP/EREBP (Os05g0121600), MYC-related bHLH (Os07g0143200), and two zinc finger-related TFs (Os12g0581900, Os05g0102200). In addition to TFs, the non-TF homologs of calmodulin (Os03g0743500), β-glucosidase 11 (Os09g0511600), three heatshock proteins (one homolog of Os03g0271350 and two homologs of Os02g0128400) were detected.

**Group II**: Group II was also characterized by acute response, showing up-regulation in 5 min followed by down-regulation in 30 min. Group II was composed of 33 genes (S3 Table), including three MYB domain proteins (one homolog of Os02g0194000 and two homologs of Os07g0119300). Additionally, as non-TF homologs, calcium-binding EF-hand family protein (Os09g0412300), phospholipase C (Os09g0535900), and *LOW EXPRESSION OF OSMOTICALLY RESPONSIVE GENES 1* (*LOS1*; Os04g0118400), a known translation elongation factor 2-like protein [38].

**Group III**: Group III had no significant expression changes in 5 min before up-regulation occurred at 30 min. Composed of 96 genes (S3 Table), this was the largest group. It included seven TFs, including the KANADI family of putative TF (Os08g0160300), basic helix-loop-helix 105 (Os02g0116600), GATA transcription factor 24 (Os03g0684000), DHHC-type zinc finger family (Os01g0844400), homeodomain-leucine zipper TF (Os03g0109400), B-box type zinc finger TF (Os07g0667300), and basic helix-loop-helix TF (Os01g0108600). In addition, the homologs of two pathogen-related genes (PR proteins Os01g0899800 and Os04g0593400) and cell damage protection and recovery related genes (e.g., *BAX INHIBITOR 1* (*BI1*) (Os02g0125300) [39], *ACCELERATED CELL DEATH 5* (*ACD5*) (Os02g0656200) [40], *RECOVERY PROTEIN 3* (*REV3*) (Os11g0186400) [41], and *ASYNAPTIC 1* (*ASY1*) (Os03g0202800) [42]). Also, a known epigenetic regulator, *REPRESSOR OF SCILENCING 3* (*ROS3*) [43], was detected in Group III.

**Group IV**: Group IV had no significant change in 5 min, but down-regulation of expression occurred in 30 min, and formed 22 genes (S3 Table). Of them, two TFs (*AUXIN RESPONSIVE FACTOR* (Os02g0628600) [44] and DNA binding TF-encoding gene (Os03g0174900)) were identified.

**Group V**: Group V exhibited down-regulation in 5 min, followed by up-regulation in 30 min. It included 71 genes (S3 Table) with one TF (zinc-knuckle family (Os01g0715000)) five protein kinases (one homolog of Os01g0917500, one homolog of Os10g0562500, two homologs of Os06g0168800, and one homolog of Os04g0598800). This group also contained two epigenetic-related genes *HISTONE DEACETYLASE 3* (Os05g0597100) [45] and chromatin-related (Os11g0545600), and a circadian clock gene, *TOC1* (Os02g0618200) [46].

**Group VI**: Group VI exhibited initial down-regulation in 5 min and no significant changes until 30 min. This group includes 29 genes (S3 Table) with six TFs, AGL19 (Os10g0536100), two WRKY3 (Os01g0665500) homologs, zinc-finger protein (Os08g0471900), bHLH TF (Os11g0601650), *ABSCISIC ACID RESPONSIVE ELEMENTS-BINDING FACTOR 2* (Os06g0211200) that encodes ABA binding TF [47]. A histone modification gene encoding H3K4-specific methyltransferase SET7/9 family protein (Os09g0453900) is also in this group [48].

### Acute and follow-up responses and their compositions

**Fig 5:**
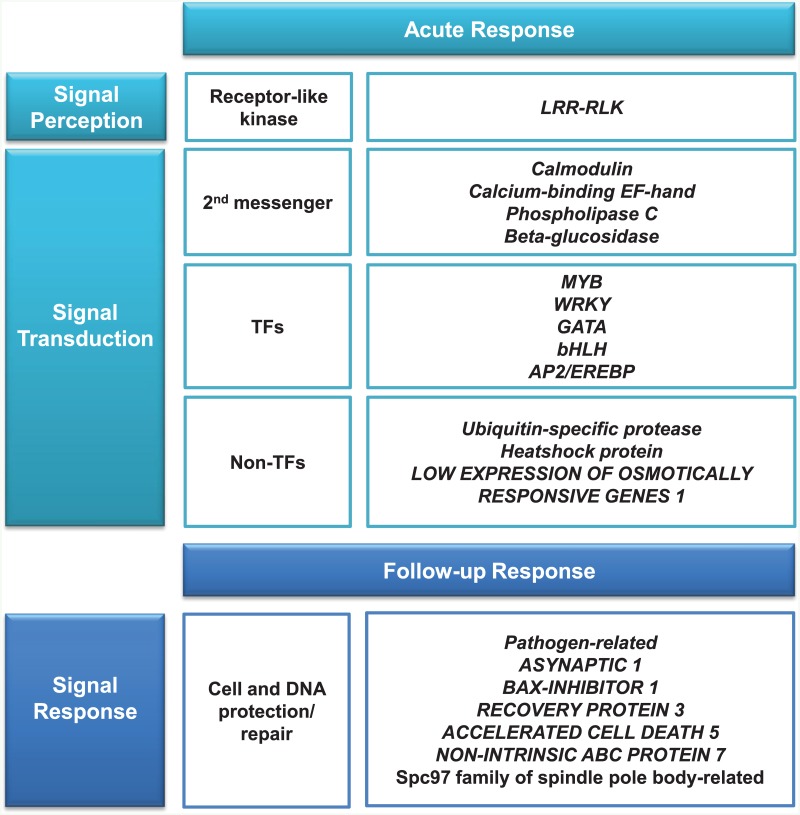
Comparison of DEGs belonging to acute and follow-up responses under freezing stress with the components of known abiotic signal transduction.

Based on GO categories of DEGs in six groups (Fig 2), several of the biological process groups (response to stress, signal transduction, transcription, metabolic process, and DNA and RNA metabolism) were extensively analyzed (S4 Table). The expression patterns of the majority of these genes featured (1) acute response (up-regulated within 5 min; Group I and II), and (2) follow-up response (up-regulated from 5 min to 30 min; Group III and V). The annotation data showed that genes belonging to the these groups were very diverse and included those that encode abiotic stress- responsive TFs, receptor kinases, stress proteins, ubiquitin-mediated proteases, hormone-responsive proteins, metabolism-related proteins, and chromatin modification-related proteins. Interestingly, the expression dynamics of these groups based on time-course reflected a logical overview of freezing signal transduction cascade: initiation of lipid metabolic and osmotic responsive genes, calmodulin, MYB TF (which affects ABA-signaling), WRKY (which affects AFP-expression), β-glucosidase at initial stage (0–5 min), followed by heat-shock protein (HSP), proteasome-related, PR protein, and cell/DNA damage repair gene expression at the following stage (5–30 min). We also detected chromatin-modification genes and circadian clock genes with dynamic expression pattern. Based on our result, we displayed a tentative gene expression order and reported genes involved in early response to freezing exposure (Fig 5).

### Validation of DEGs by real-time RT-PCR

**Fig 6:**
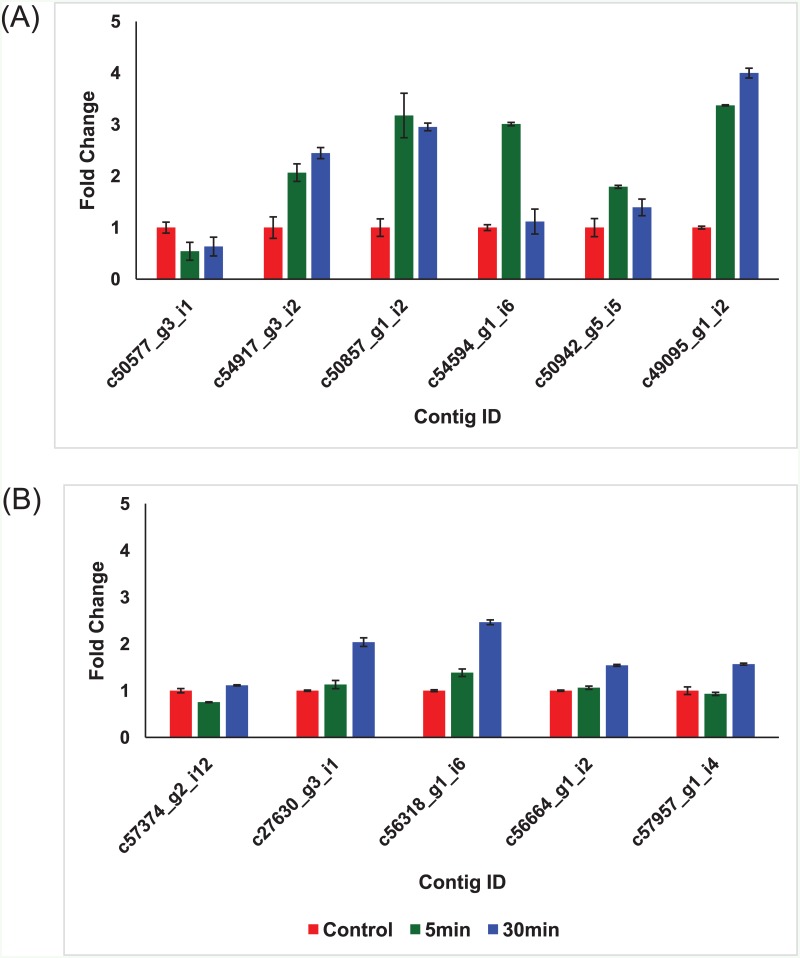
qRT-PCR validation of selected DEGs. The fold changes of expression level in 5 min and 30 min after the freezing treatment in comparison with the control (0 min). (A) qRT-PCR validation of six transcription factors from DEGs (B) qRT-PCR validation of five non-transcription factors from DEGs.

We validated 11 genes from RNA-sequencing using real-time RT-PCR analysis with actin as the reference gene that strongly correlated with those from RNA-sequencing data (Pearson correlation coefficients r = 0. 8829) (S5 Fig and S5 Table). Of these genes, 6 were TFs [bZIP TF (c50577_g3_i1), WRKY74 TF (c54917_g3_i2), GATA TF (c50857_g1_i2), MYB (c54594_g1_i6), ARF10 (c50942_g5_i5), and bHLH TF (c49095_g1_i2)] (S3 Table), and 5 were non-TFs [phosphatidate cytidylyltransferase (c57374_g2_i12), hydrolase (c27630_g3_i1), zinc-binding peroxisomal integral membrane protein (c56664_g1_i2), mRNA splicing factor (c57957_g1_i4), and unknown protein (c56318_g1_i6)] (S3 Table). Five out of 6 TF-encoding genes showed rapid up-regulation in 5 to 30 min (Fig 6A), while 5 non-TF-encoding genes were up-regulated in later stages (e.g., 30 min) (Fig 6B).

## Discussion

It is expected that freezing tolerance at the cellular level should be initiated by rapid signaling so that the cell can take immediate action. In this regard, we examined the freezing responsive gene expression in prairie cordgrass based on hierarchical clustering of expression pattern changes from 0 to 5 min and 5 to 30 min. Because we were unable to determine that down-regulation or negatively regulated signaling had a damaging effect, we mainly focused on up-regulated gene expression patterns. We could detect the potential components that responsive to freezing stress and the elaborate coordination between DEGs from two major responded se groups (acute and follow-up) and the signal transduction pathway.

### Acute response group is characterized by metabolic, transcriptional, and post-translational components of signal transduction

The acute response is critical for cell survival under extremely low temperatures. Interestingly, early responses were characterized by the acute reaction of rapid gene up-regulation without significant changes or downshifting until 30 min (Group I and II). This implies that their immediate upstream action is in signal perception and/or transduction. First, we detected genes that encode plasma membrane-localized components (lipid metabolism-related enzymes), phospholipase C (PLC), and osmotically responsive proteins (LOS1)). When exposed to freezing, a low temperature signal has to be perceived and transmitted to the nucleus. In this aspect, the components located in the cell membrane must first detect and amplify the signal. Phospholipase C, a major lipid hydrolyzing enzyme, is involved in lipid-mediated signaling [49],is known to be an ABA-related protein, and considered as an ABA receptor within the ABA signaling pathway [50,51]. It has been reported that in cold-treated *Arabidopsis*, the PLC pathway was activated and served as an upstream cold response signal [52]. The cold-treated transcriptome changes were investigated using the agents that modified PLC and revealed that the expression of many genes was modified during the cold response, particularly through the CBF-mediated pathway [52]. In *Arabidopsis thaliana* and *Vigna radiate* (mungbean), most PLC paralogs were highly up-regulated in response to several different abiotic stresses, such as cold, drought, and salt [49,53]. In *Oryza sativa*, transcript profiling using microarray showed that most of the PLC paralogs were expressed differentially under cold, drought, and salt stresses at various developmental stages [54]. LOS1 is a translation elongation factor 2-like protein. It is involved in cold-responsive translation which plays an important role in freezing signal transduction [38]. Previous findings showed that *los1-1* mutants failed to acclimate to cold stress and became freezing-sensitive [55]. Therefore, the significant up-regulation of components in cell membrane occurs at an early stage because it serves as an important signal transducer and is known to be inducible in response to ABA and ROS, salt, and drought stresses [29]. Moreover, significant up-regulation of cell membrane components resulting in acute responses may play initial roles in signal perception and transduction at the early stages of freezing.

β-glucosidase is known to be membrane-associated and is the key enzyme that mediates the conversion of cellobiose to glucose during cellulose hydrolysis [30, 56], There has been little study of β-glucosidase related to abiotic stress, but the constitutive up-regulation of β-glucosidase in a flood-tolerant *Echinochloa* accession [57] and freezing tolerance in *Arabidopsis* transgenic lines of *SRF2* (*SENSITIVE TO FREEZING2*), which encodes β-glucosidase [58], have been reported.

Transcription factors, particularly MYB and WRKY family members, play pivotal roles in early response in tolerance signal cascade. The *MYB* gene is involved in a wide range of abiotic stresses and ABA signaling [59,60]; in *Arabidopsis*, *MYB* is involved in cold stress, regulates ABA-responsive genes, and plays an important role in the upstream step of cold stress signal transduction. In addition to *MYB*, it has also been reported that under severe osmotic/salt stress, the over-expression of *WRKY46* in *Arabidopsis* mediated later root development through regulation of ABA signaling [61]. The *WRKY* gene family is involved in multiple stresses [62], playing important roles in abiotic stress tolerance and ABA-signaling [63]. Specifically, WRKY is known to promote the expression of PR-protein or anti-freezing protein (AFP) in response to abiotic stress [64,65]. PR-protein is also known to act as an AFP, arresting ice-crystallization in apoplasts [64]. Once water molecules located in the apoplast start to freeze, which leads to drought or osmotic stress condition, the intracellular water will leak through the cell membrane, resulting in the removal of water from inside the cells [64]. WRKY plays an important role in increasing up-regulation of PR gene expression [66,67], and in fact, we detected PR gene expression in the follow-up response stage, after WRKY expression in acute stage. WRKY is also known to be involved in cold, heat, drought, and salt stress responses [68]. Moreover, it was shown that over-expression of one of WRKY family members, WRKY44 acquired multiple abiotic stress tolerance [62] in tobacco. Our data showed that WRKY acts as a potential upstream regulator in freezing or may be involved in tolerances to multiple stresses.

We also detected the calcium-signaling components, calmodulin 1 homolog, which acts as an intracellular calcium sensor in cold, drought, and salt [69] stresses. The up-regulation of camodulin indicates that calcium-mediated signaling is involved in freezing tolerance. Calcium-binding EF-hand family protein is also known to be involved in salt and drought-stress tolerance mechanisms [70,71]. The up-regulation of these genes indicates that calcium signaling is also important in freezing signal transduction.

The presence of an ubiquitin-mediated proteasome component indicates that post-translational regulation was also actively involved in abiotic stress tolerance [72,73]. For example, the target of a negative regulator for degradation under abiotic stress by ubiquitin ligase may initiate the action of signaling for stress tolerance. Alternately, ubiquitin-mediated degradation of positive regulators may repress the action for tolerance until the stress signal is received. Regulation of the ubiquitination pathway for abiotic stress tolerance in rice, implied that its application to crops may improve stress tolerance [74]. Although we did not identify the target of ubiquitin-mediated proteolysis, we recognize the importance of protein degradation as a regulatory mechanism of freezing response.

### Follow-up response components consist of genes involved in cell protection and DNA recovery mechanism

The follow-up response group was characterized by genes that encoded PR-proteins and DNA-damage repair proteins. Pathogen-Related genes (PR-protein) can play dual roles in biotic and abiotic stress tolerance. This PR-protein may serve as a stress protein by attaching to ice crystals in order to inhibit increase of ice crystal formation, as a role of anti-freezing protein (AFP). At low temperatures, apoplastic AFP in winter rye had sequences and function similar to PR protein [64]. Griffith et al. [65] also reported that plant AFPs carry multiple domains for ice-binding and these AFPs are homologous to PR proteins that protect plants against pathogen. In addition, the follow-up response group was characterized by several genes that encode DNA repair proteins (RECOVERY PROTEIN 3 (REV3)). This is a catalytic subunit of DNA polymerase that following mutation, exhibited sensitivity to UV-B radiation and is involved in damage-tolerance mechanisms through translesion synthesis [41,75]. Acting as an attenuator of biotic and abiotic stress-causing programmed cell death is BAX-INHIBITOR 1 (BI1) [39,76]; NON-INTRINSIC ABC PROTEIN 7 is involved in biogenesis and/or repair of oxidatively damaged Fe–S clusters [77] and ASYNAPTIC 1 is involved in DNA repair during meiosis [42]. Therefore, it appears that cell protection and DNA repair mechanisms were highly activated at follow-up stages.

### Epigenetic-related and clock genes were responsive to freezing stress

In Groups I, III, V, and VI, we found 5 chromatin modification-related genes and a clock gene, indicating dynamic action of chromatin modification in response to freezing. In Group I, SWIRM expression was found as an acute response. SWIRM is a component of the SWI/SNF and RSC chromatin remodeling complexes and known to be responsive to abiotic stress [78]. In rice, the genes encoding SWIRM domain proteins were induced by heat stress and reduced by cold stress. Chromatin modification in response to abiotic stress (drought, heat, cold, and salt) was recently studied, and showed increased involvement in stress tolerance mechanism [79]. Considering that Group I is characterized by acute up-regulation, an immediate SWIRM response may play an important role in freezing response. REPRESSOR OF SCILENCING 3 (ROS3), a RNA-binding protein required for DNA demethylation that leads to transcriptional gene activating in *Arabidopsis* [43] and was detected in Group III, indicating that this gene acted in follow-up response via transcriptional regulation of downstream genes. In Group V, a recessive mutation of *HDA19*, HISTONE DEACETYLASE, was hypersensitive to ABA and salt stress in *Arabidopsis* [45,80]. The initial down-regulation of HDA can be as rapid as 5 min and the damaged cell repressed HDA’s negative role in gene expression, possibly due to enhanced stress sensitivity. Conversely, while up-regulation of HDA after 5 min restored negative actions, identifying the HAD target genes could provide clarity. In Group VI, H3K4-specific methyltransferase was detected; its over-expression increased salt tolerance in *Arabidopsis* [81]. Moreover, it was reported that ABA or abiotic stress treatments enhanced H3K4 trimethylation of stress-responsive genes [48]. In our study, the H3K4-specific methyltransferase gene was down-regulated within 5 min. Its target genes need identification to explain this pattern. In Group V, we identified TIMING OF CAB EXPRESSION 1 (TOC1), a major component of the circadian clock and known to be regulated by chromatin modification. Because it can also bind to the promoter region of ABA-related genes (*ABAR*, *CHLH*, *GUN5*) and control circadian expression [46], TOC1 appears to be related to abiotic stress tolerance. It was reported that TOC1 and ABAR were over-expressed and that RNAi plants had defective responses to drought, implying the importance of clock-dependent ABA function in drought stress [46]. In fact, many ABA-related genes regulated by the circadian clock have been reported [82–84].

### DEGs and abiotic stress signaling pathway

We also found that acute-response DEGs corresponded with genes for signal perception and transduction, while follow-up response DEGs corresponded strongly with signal-response genes (Fig 5). According to Winfield [29], the perception of cold stimulus may be sensed through membrane modification such as fluidity or rigidity [85–87]. For example, a membrane-bound kinase, such as the receptor-like kinase (RLK) in our analysis, may be responsible for upstream signaling cascade as a consequence of the mechanical modification of plasma membrane or other factors. The perceived signal is then transmitted to next step via second messengers, calcium-binding proteins (CBPs) for example, followed by the activation of kinases and/or phosphatases, which in turn regulate the transcription of TFs in the nucleus. We also found the calcium-binding proteins, calmodulin and Ca^2+^-binding EF hand, which are known to act as second messengers. Calcium-signaling appears to be important in freezing tolerance response. Once the signal is transferred to nucleus, TFs that regulate stress-responsive proteins are activated. These TFs include WRKY, an activator of genes that encode AFPs; MYB, an activator of sugar/SOD pathway; and ICE1, an activator of stress proteins such as COR/LEA [87,88]. We did not find many of the cold-stress responsive genes, (e.g., *CBF*, *CDPK*, *COR*, *ERD*, or *RD*) [87–91], suggesting that the freezing-response pathway may be different than the cold response pathway. Instead, we found *MYB*, *WRKY*, *GATA*, *bHLH*, and *AP2/EREBP*, which are known to be related to abiotic stress responses such as cold, drought, and salt. Thus, these TFs share in freezing stress tolerance. In fact, we found up-regulation of the PR gene after the acute response of *WRKY* expression, indicating that WRKY-controlled AFP (PR) genes in response to freezing. We also detected the signal-response genes, *ASYNAPTIC 1*, *BAX-INHIBITOR 1*, *RECOVERY PROTEIN 3*, *ACCELERATED CELL DEATH 5*, and *NON-INTRINSIC ABC PROTEIN 7*, known to be cell and DNA repair genes. In this work, we detected the rapid and well-coordinated serial induction of DEGs that exhibited induction of signal perception and transduction-related gene expression changes, followed by signal-response gene expression change, which reflects the process of signal-transduction cascade in response to abiotic stress. In addition to signaling components, we found metabolic and epigenetic components that may play important roles in early responses to freezing, which require further study. Freezing-responsive DEGs discovered from *S*. *pectinata* transcriptomes can facilitate the identification of freezing-tolerance genes and serve as useful sources for future functional studies and improving the freezing tolerances of crop varieties.

## Supporting Information

S1 Fig Screening of freezing tolerance in prairie cordgrass under three freezing temperatures with six time duration. (PDF)

S2 Fig Workflow of bioinformatics process. (PDF)

S3 Fig Freezing treated two genotypes of prairie cordgrasses *S*. *pectinata* "Savory" and *S*. *pectinata* "17–109"; and two switchgrasses *P*. *virgatum* var. "Kanlow" and *P*. *virgatum* var. "Cave-in-Rock; A; No treatment B; Sub-zero -5°C; treatment. (PDF)

S4 Fig Variation in electrolyte leakage response of three C_4_ grasses under sub-zero treatment 0°C to -7°C. (PDF)

S5 Fig Correlation test between RNAseq and qRT-PCR. (PDF)

S1 Table Primers used for qRT-PCR validation of selected DEGs. (XLSX)

S2 Table Annotation of prairie cordgrass leaf transcriptome against Plant RefSeq protein database. (XLSX)

S3 Table Annotation of six DEG groups. (XLSX)

S4 Table Five major GO categories of biological process in relation to abiotic stress responses. (XLSX)

S5 Table Correlation test for individual genes used for qRT-PCR validation. (XLSX)
