# Supplementary figures and images for: Transcriptome Analysis of Spartina pectinata in Response to Freezing Stress
Source: PLoS One. 2016 Mar 31;11(3):e0152294. doi: 10.1371/journal.pone.0152294 (PMC4816275; doi:10.1371/journal.pone.0152294)

# Prairie cordgrass freezing tolerance

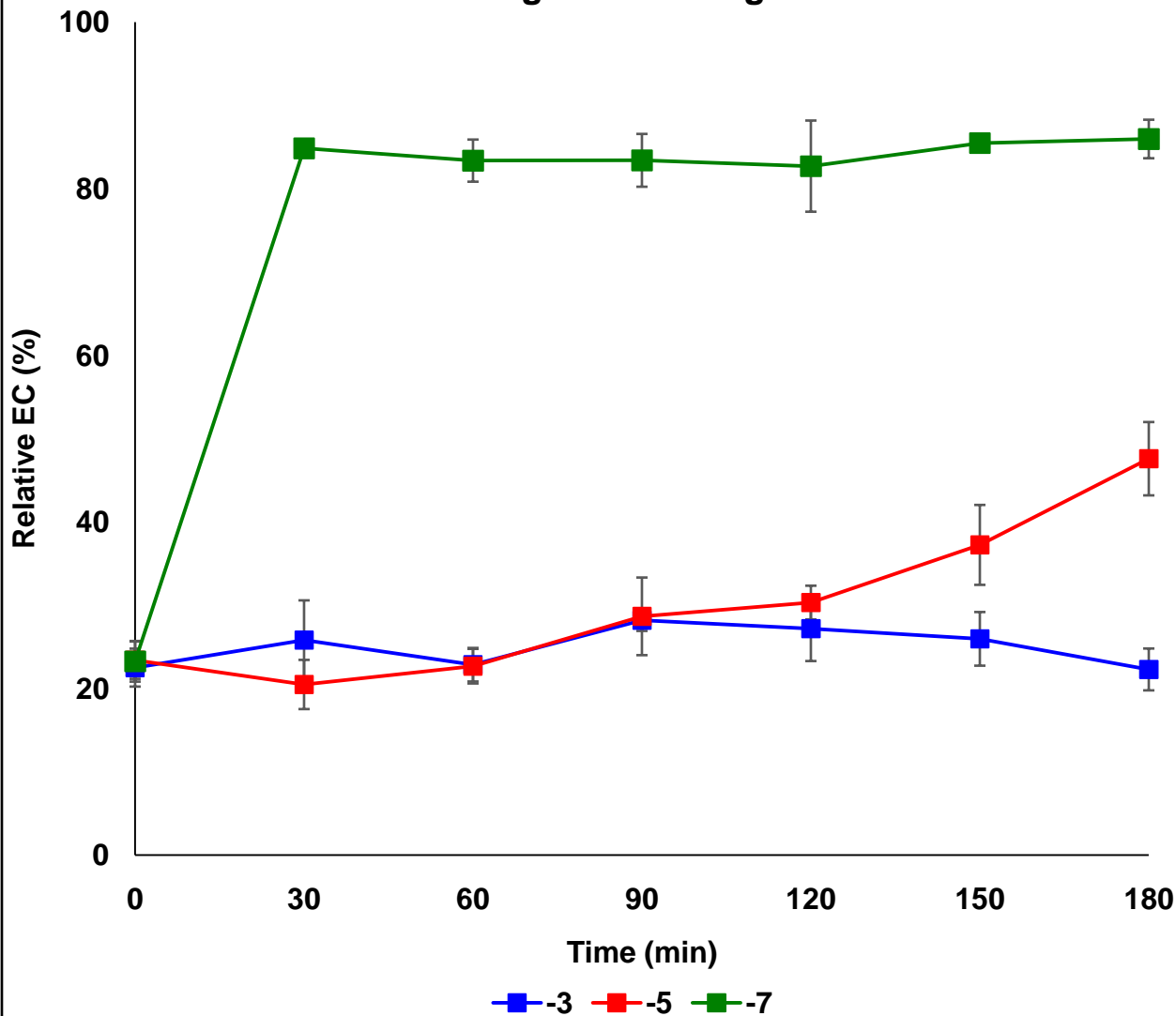

Supplement: S1 Fig — (PDF) [file pone.0152294.s001.pdf]

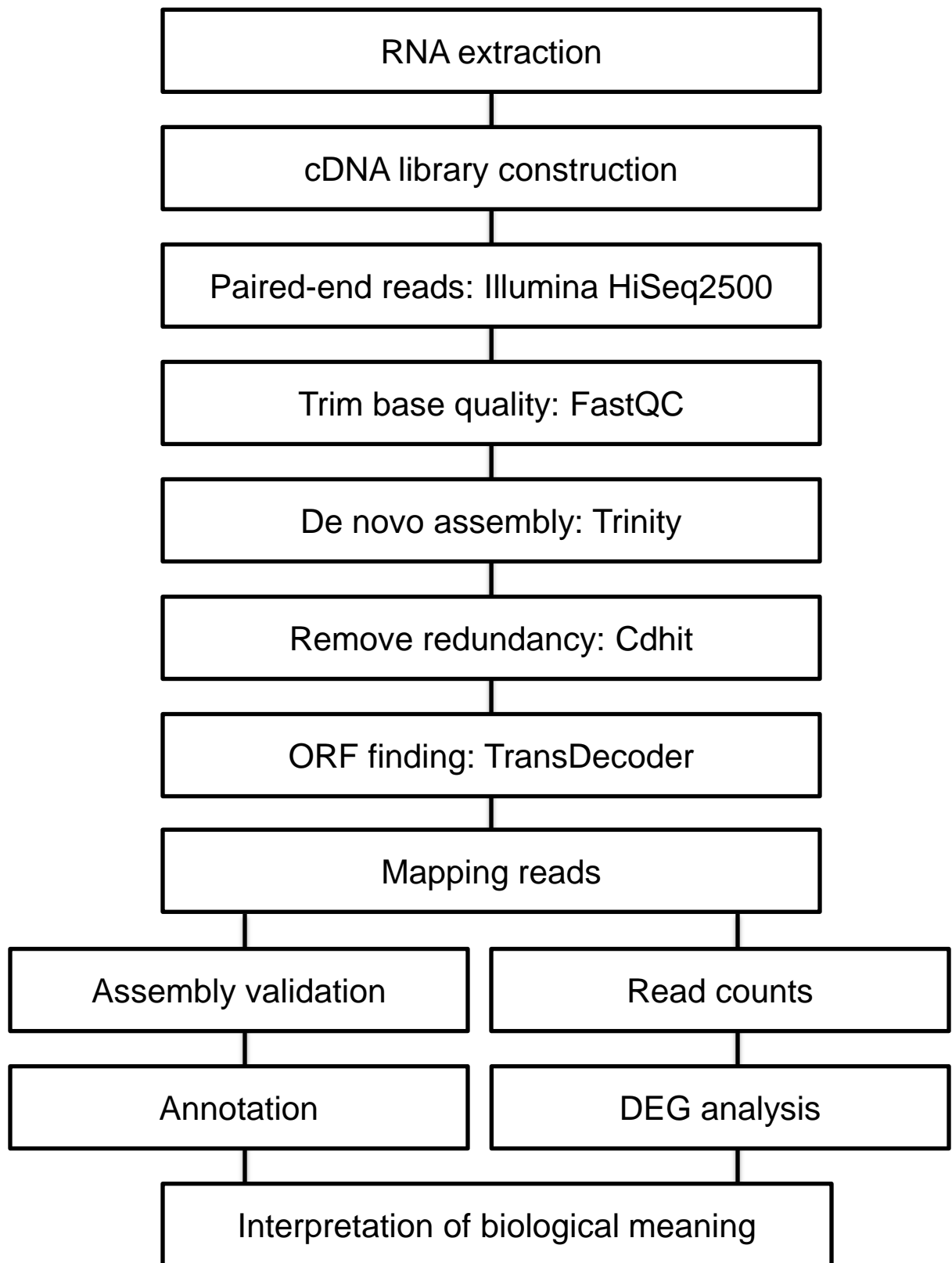

Supplement: S2 Fig — (PDF) [file pone.0152294.s002.pdf]

(A)

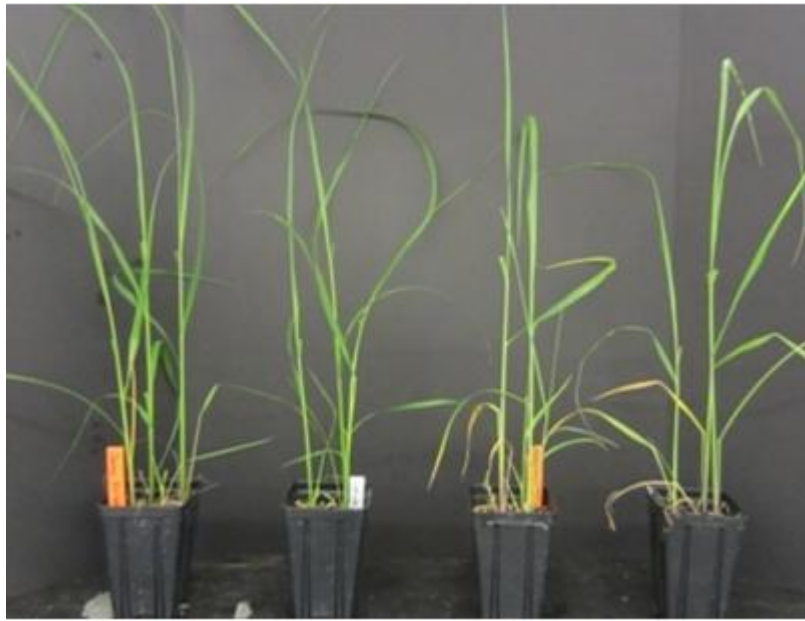

(B)

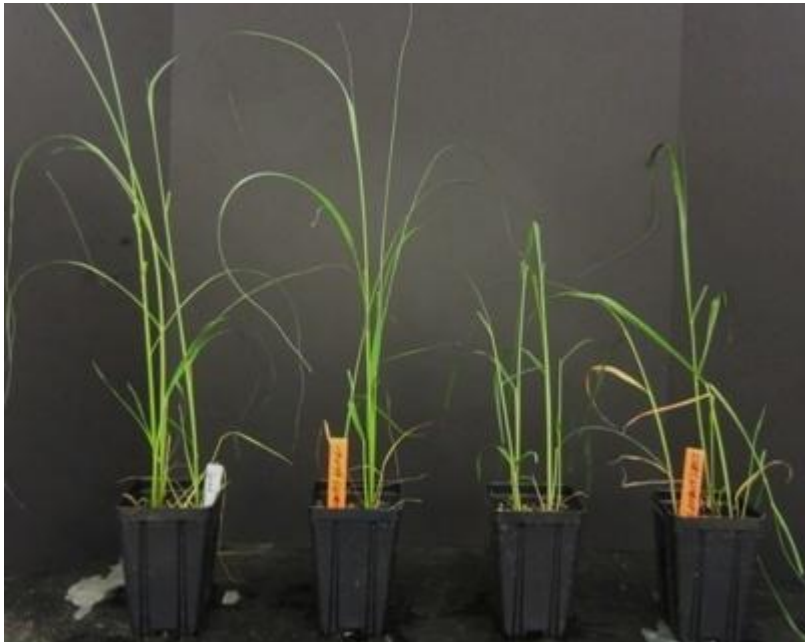

*S. pectinata* "Savory"

*S. pectinata* "17-109"

*P. virgatum* var. "Kanlow"

*P. virgatum* var. "Cave-in-Rock"

Supplement: S3 Fig — (PDF) [file pone.0152294.s003.pdf]

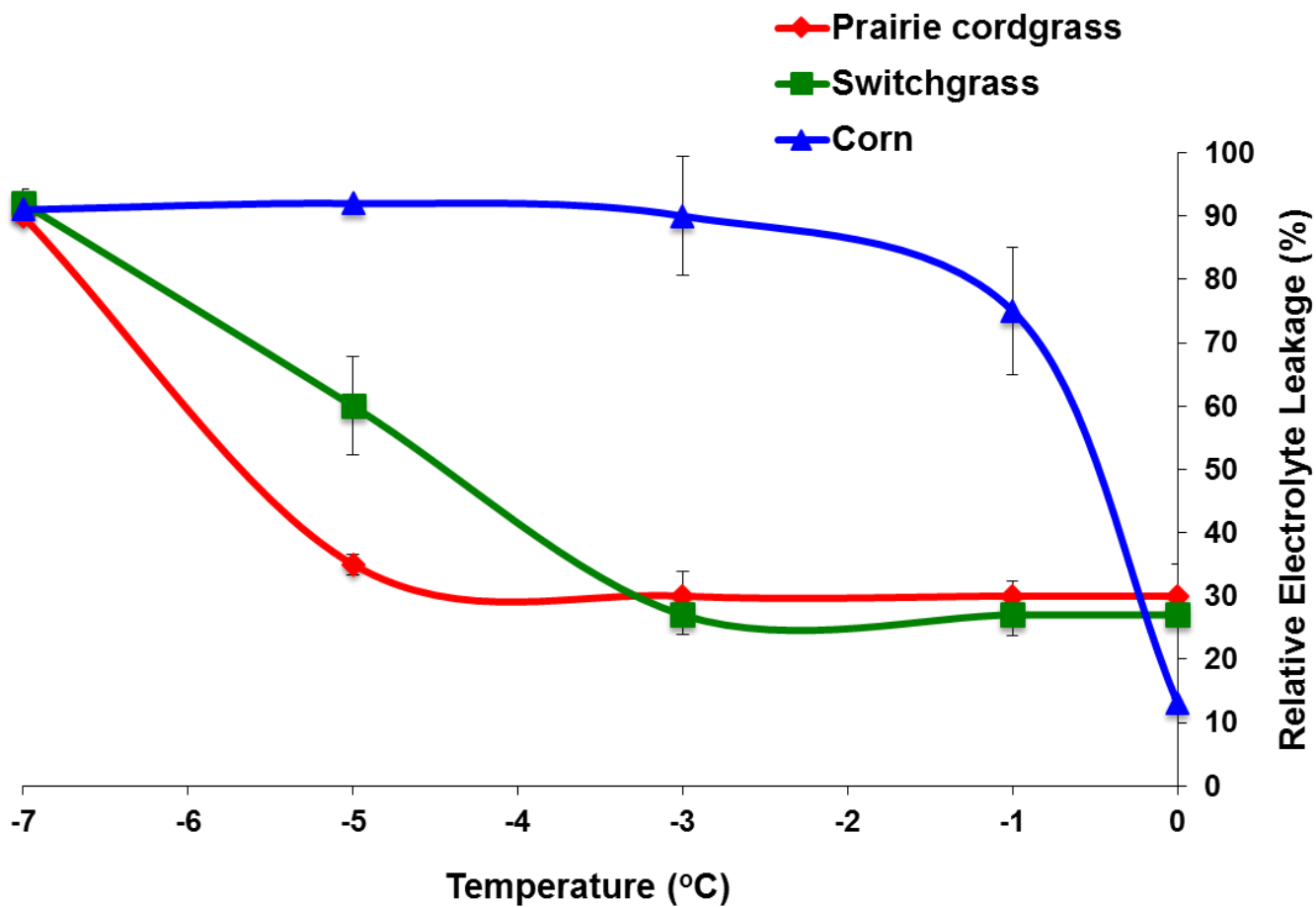

Supplement: S4 Fig — (PDF) [file pone.0152294.s004.pdf]

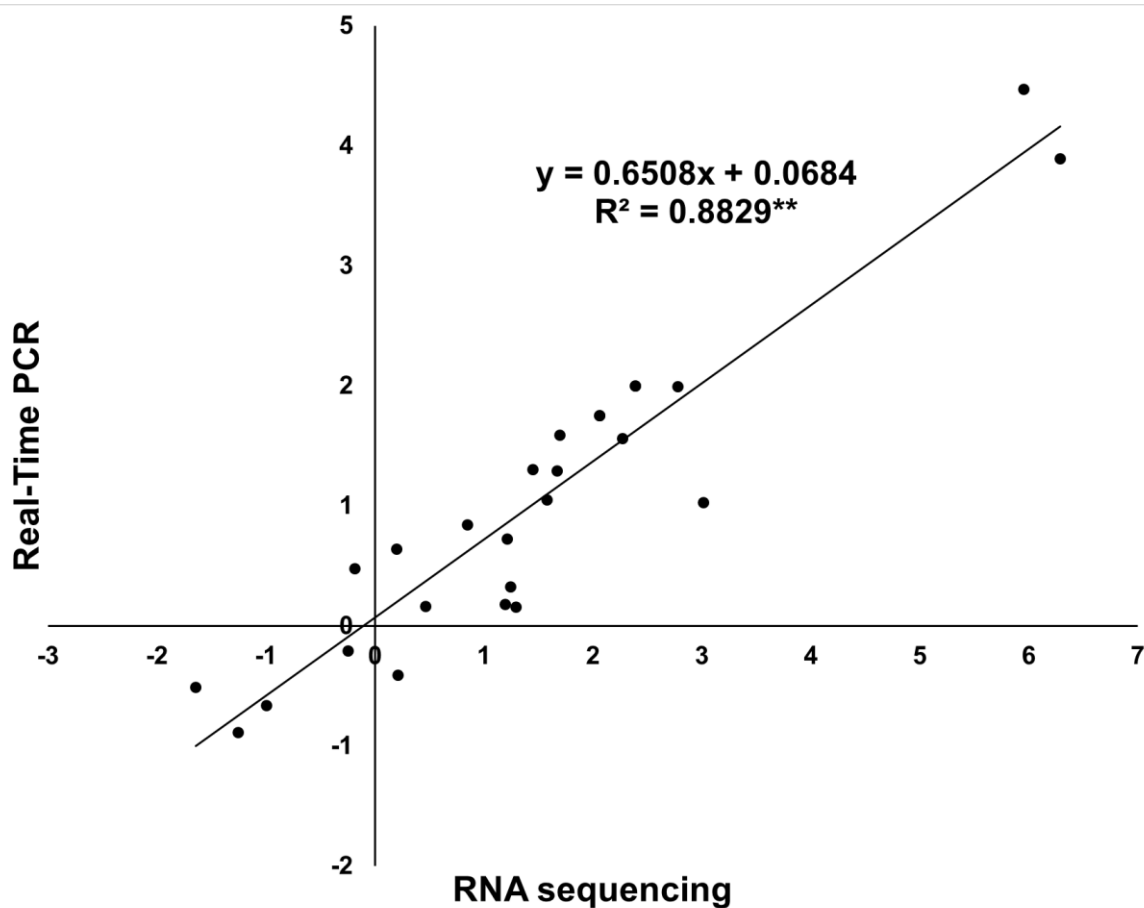

Supplement: S5 Fig — (PDF) [file pone.0152294.s005.pdf]
